# The myofibrillar myopathy–linked variant *DES*-p.T341P impairs desmin filament assembly

**DOI:** 10.1007/s11033-026-11696-z

**Published:** 2026-03-25

**Authors:** Alexander Lütkemeyer, Sabrina Voß, Franziska Klag, Joline Groß, Jonas Reckmann, Anna Gärtner, Dario Anselmetti, Jan Gummert, Volker Walhorn, Hendrik Milting, Andreas Brodehl

**Affiliations:** 1 Heart and Diabetes Center NRW, Georgstrasse 11, 32545 Bad Oeynhausen, Ruhr University Bochum, Bochum, Germany; 2 Faculty of Physics, Experimental Biophysics & Applied Nanoscience, Universitätsstrasse 25, 33615 Bielefeld, Bielefeld University, Bielefeld, Germany

## Abstract

**Background:**

Desminopathies are clinical heterogenous and range from isolated skeletal myopathies to different cardiomyopathies or combinations of both. At the molecular level, an aberrant cytoplasmic desmin aggregation is a typical hallmark of pathogenic *DES* variants. Currently, it is difficult to predict an aberrant desmin aggregation of novel *DES* variants without functional analysis.

**Methods:**

Therefore, we investigate in this study the impact of an uncharacterized myofibrillar myopathy-associated desmin variant (p.T341P) on filament assembly in transfected cells and performed atomic force microscopy to characterize in vitro the filament assembly of recombinant desmin-p.T341P.

**Results:**

Based on these cell transfection experiments using different cell lines and cardiomyocytes differentiated from induced pluripotent stem cells (iPSC) we present evidence that the filament assembly of desmin-p.T341P is disrupted. In addition, atomic force microscopy (AFM) analysis revealed aberrant molecular structures of recombinant desmin in comparison to wild-type desmin. These experiments showed an intrinsic filament assembly defect of desmin-p.T341P. Therefore, we suggest to classify this *DES* variant as a likely pathogenic variant associated with myofibrillar myopathy and heart failure.

**Conclusion:**

In conclusion, functional analysis of the desmin filament assembly can contribute to the pathogenicity classification of further *DES* variants and may be helpful in genetic counselling of affected patients.

**Supplementary Information:**

The online version contains supplementary material available at 10.1007/s11033-026-11696-z.

## Introduction

Rare genetic variants in the *DES* gene (OMIM, *125660) cause different cardiac and skeletal myopathies, including dilated, restrictive or arrhythmogenic cardiomyopathy and myofibrillar myopathy (MFM) [1–3]. MFM is a genetically heterogeneous group of muscle disorders characterized by progressive disruption of myofibrils and abnormal cytoplasmic protein aggregation within myocytes (OMIM, #601419) leading to progressive muscle weakness in combination with cardiomyopathy [4–6]. The *DES* gene encodes the muscle-specific intermediate filament (IF) protein desmin, which connects different multi-protein complexes and cell organelles like the Z-bands, cardiac desmosomes and mitochondria in cardiomyocytes [7–9]. Therefore, desmin is highly important for the structural integrity of cardiomyocytes [10, 11]. Recently, the novel *DES* variant p.T341P (c.1021 A > C) was described in a small Russian family with MFM including heart failure [12–14]. However, functional data to this specific *DES*-p.T341P variant are currently missing.

Therefore, we addressed here if the desmin filament assembly is affected by this variant.

## Materials and methods

### Plasmid generation

The generation of the wild-type desmin encoding plasmids pEYFP-N1-DES and pET100D-TOPO-DES have been previously described [15]. We inserted the variant p.T341P using the QuikChange Lightning Kit (Agilent Technologies, Santa Clara, CA, USA) in combination the two following oligonucleotides 5’-ACGCCCTCAAGGGCCCTAACGATTCCCTG-3’ and 5’-CAGGGAATCGTTAGGGCCCTTGAGGGCGT-3’ (Microsynth, Balgach, Switzerland) to generate pEYFP-N1-DES-p.T341P and pET100D-TOPO-DES-p.T341P. The *DES*-sequences of all plasmids were verified by Sanger sequencing (Macrogen, Amsterdam, Netherlands).

### Cell culture

SW-13 and H9c2 cells were cultured under standard conditions (5% CO_2_, 37 °C) in Dulbecco’s Modified Eagle Medium (Thermo Fisher Scientific, Waltham, MA, USA) supplemented with 10% fetal calf serum Superior (S0615, Sigma-Aldrich). The induced pluripotent stem cell (iPSC) line NP00040-8 (UKKi011-A, https://ebisc.org/UKKi011-A), generated from a healthy donor, was kindly provided by Dr. Tomo Šarić (University of Cologne, Germany) and were cultured in Essential 8 medium (Thermo Fisher Scientific) on vitronectin-coated culture plates.

### Differentiation of induced pluripotent stem cells into cardiomyocytes

Differentiation of iPSC into cardiomyocytes was performed as previously explained in detail [16]. Afterwards, cardiomyocytes were selected for five days using glucose-free RPMI 1640 medium (Thermo Fisher Scientific) supplemented with 4 mM sodium lactate. The iPSC-derived cardiomyocytes were maturated for more than hundred days as previously described [16].

### Transient cell transfection

Lipofectamin 3000 (Thermo Fisher Scientific) was used according to the manufacturer’s instruction for transient cell transfection. 200 ng plasmid DNA were used for SW-13 and H9c2 cells and 750 ng for iPSC-derived cardiomyocytes per well (1 cm^2^ growth area, 100,000 cells).

### Fixation and staining

Cells were washed 16 h after transfection with phosphate buffered saline (PBS; Thermo Fisher Scientific). Afterwards, the cells were fixed with 4% Histofix (Carl Roth, Karlsruhe, Germany, 15 min, room temperature, RT) and were permeabilized using 0.1% Triton-X-100 (15 min, RT). Phalloidin conjugated with Texas-Red (Thermo Fisher Scientific, 1:400, 40 min, RT) and 4’,6-diamidino-2-phenylindole (DAPI; 1 µg/mL, 5 min, RT) were used to stain F-actin and the nuclei. In iPSC-derived cardiomyocytes, α-actinin-2 was stained using mouse anti-α-actinin-2 antibodies (1:200, A7732, Sigma-Aldrich, Burlington, MA, USA) in combination with goat anti-mouse immunoglobulin G antibodies conjugates with Alexa Fluor 568 (1:200, A11004, Thermo Fisher Scientific).

### Confocal microscopy

The SP8 confocal microscope (Leica Microsystems, Wetzlar, Germany) was used for confocal microscopy as previously described in detail [17]. Deconvolution was applied for analysis using the Huygens Essential software (Scientific Volume Imaging, Hilversum, Netherlands). All images are shown as maximum intensity projections using the LAS X software (Leica Microsystems). Cellular filament and aggregate formation were manually evaluated by a blinded and trained investigator. Four phenotypic categories were defined (Supplementary Material, Figure S1): cells containing exclusively desmin filaments of varying size and length (‘Filaments’); cells with cytoplasmic, isolated aggregates (‘Aggregates’); cells with chain-like aggregates and cells displaying filaments and aggregates (‘Mixed Phenotype’).

### Expression analysis

For expression analysis the cells were grown and transfected in 48- (1 cm^2^ growth area, 100,000 cells) or 12-well plates (4 cm^2^ growth area, 400,000 cells; Greiner Bio-One, Frickenhausen, Germany). 16 h after transfection, the cells were washed with PBS. Mutant and wild-type desmin was expressed as a C-terminal fusion protein with EYFP. Fluorescence intensity of desmin-EYFP was measured using the Infinite M1000 plate reader (Tecan, Männedorf, Switzerland; excitation: 488 ± 10 nm; emission 520 ± 20 nm, *n* = 4). Non-transfected cells were used as negative controls. In addition, we analyzed the fluorescence intensity of transfected cells using fluorescence activated cell sorting (FACS) using the FACS Melody Cell Sorter (BD, Franklin Lakes, NJ, USA). The FLOWJO software (BD) was used for FACS analysis.

### Desmin expression and purification

*Escherichia coli* (BL21 Star DE3, Thermo Fisher Scientific) was transformed with desmin expression vectors pET100D-Topo-DES. Desmin expression and purification was performed as previously described [1].

### Atomic force microscopy

Desmin filament assembly was induced in vitro by addition of sodium chloride and AFM analysis was performed as previously described [1].

### Molecular modelling

Eibauer et al. described recently the molecular filament structure of the paralogous protein vimentin [18, 19]. We used this vimentin structure to model the desmin tetramer using the SWISS-MODELL server (https://swissmodel.expasy.org/) [20]. PyMOL Molecular Graphics Systems (Schrödinger LLC, New York, NY, USA) was used for visualization of the molecular desmin structure.

### Statistical analysis

All cell transfections were performed in quadruplicated analyzing about 100 cells per independent transfection experiment. For statistical comparison, the independent transfection experiments were used as units of analysis (*n* = 4). The GraphPad Prism 10 Software (GraphPad Software, Boston, MA, USA) was used for statistical analysis using the non-parametric Mann-Whitney test. The data are shown as dot plots. .

## Results

**Fig. 1:**
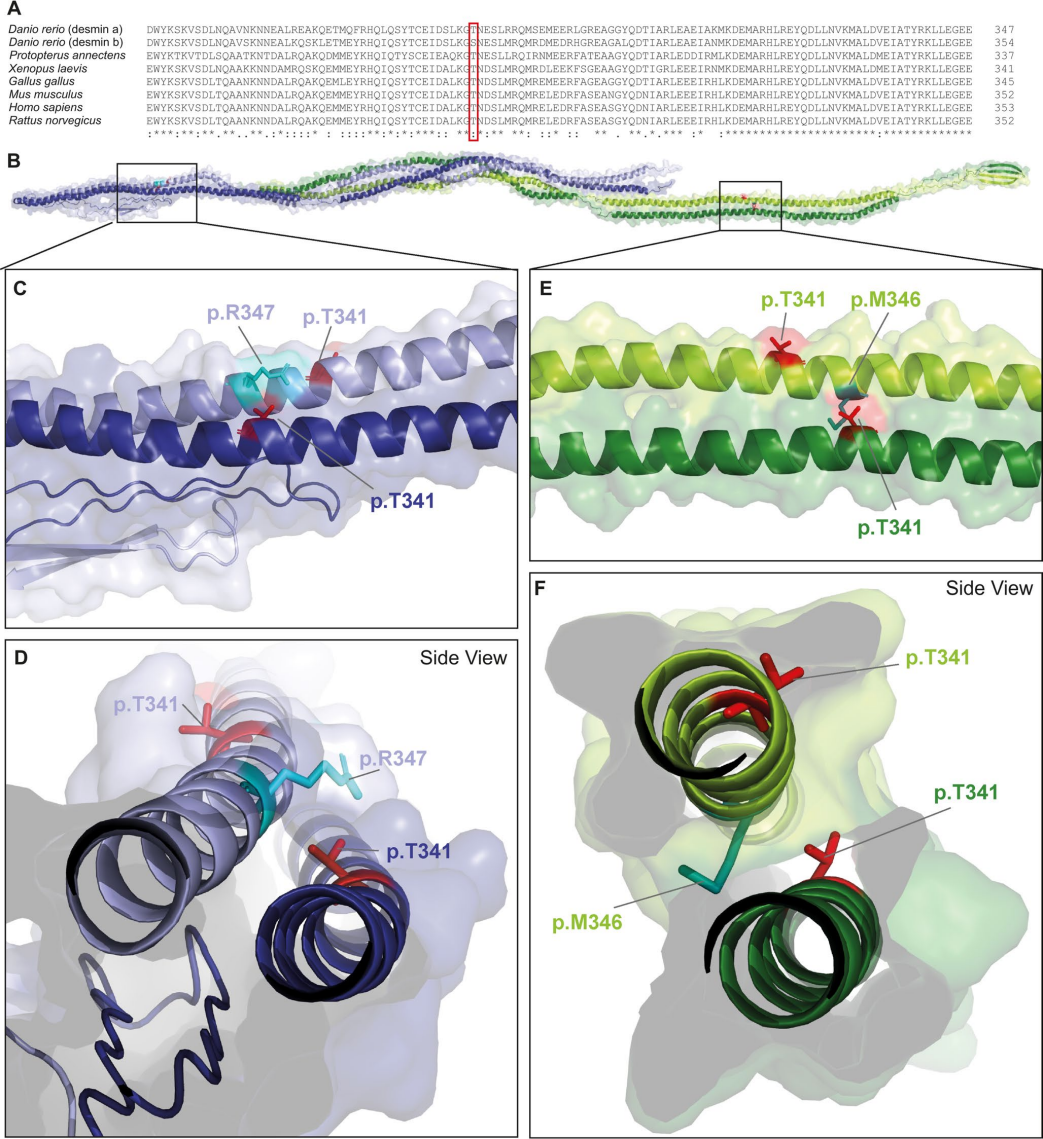
Molecular analysis of desmin-p.T341P. **(A)** Partial desmin sequence alignment of different vertebrate species. Threonine 341 is highlighted by a red box. Identical amino acids are indicated with asterisks, and similar ones are categorized with colons. Of note, threonine is conserved between most vertebrate species. The following sequences have been used for the protein alignment: NP_571038.2; NP_001070920.2; XP_043931805.1; NP_001080177.1; NP_034173.1; NP_001918.3; NP_071976.2. **(B-F)** Molecular model of the antiparallel desmin tetramer formed by two coiled-coil dimers (shown in green and blue). Threonine 341 residues are presented in red and are labelled in the color of the corresponding helices. **(C-D)** Arginine 347 (cyan) is in close proximity to threonine 341. **(E-F)** In the second region, methionine 346 (turquoise) of the parallel helix is in proximity to threonine 341

Threonine 341 is localized in the highly conserved coil-2 subdomain. In most other vertebrates a threonine residue is present at this position of desmin (Fig. 1A). Only in desmin‑b from *Danio rerio* a serine residue is present, which, closely resembles the functional properties of threonine due to its hydroxyl group (Fig. 1A). Two parallel coiled-coil desmin dimers assemble into antiparallel asymmetric tetramers (Fig. 1B). In one dimer, the C‑terminal tail domains build a relatively extended, linear configuration, whereas in the antiparallel dimer the tail domains appear partially folded back toward the rod domains. Therefore, the four threonine 341 residues are localized in two different molecular regions within the antiparallel desmin tetramer (Fig. 1B-F). In the first region, threonine 341 is in close proximity with arginine 347 of the parallel α-helix (Fig. 1C-D), whereas in the second region, one threonine residue is closely localized to methionine 346 of the parallel helix (Fig. 1E-F).

**Fig. 2:**
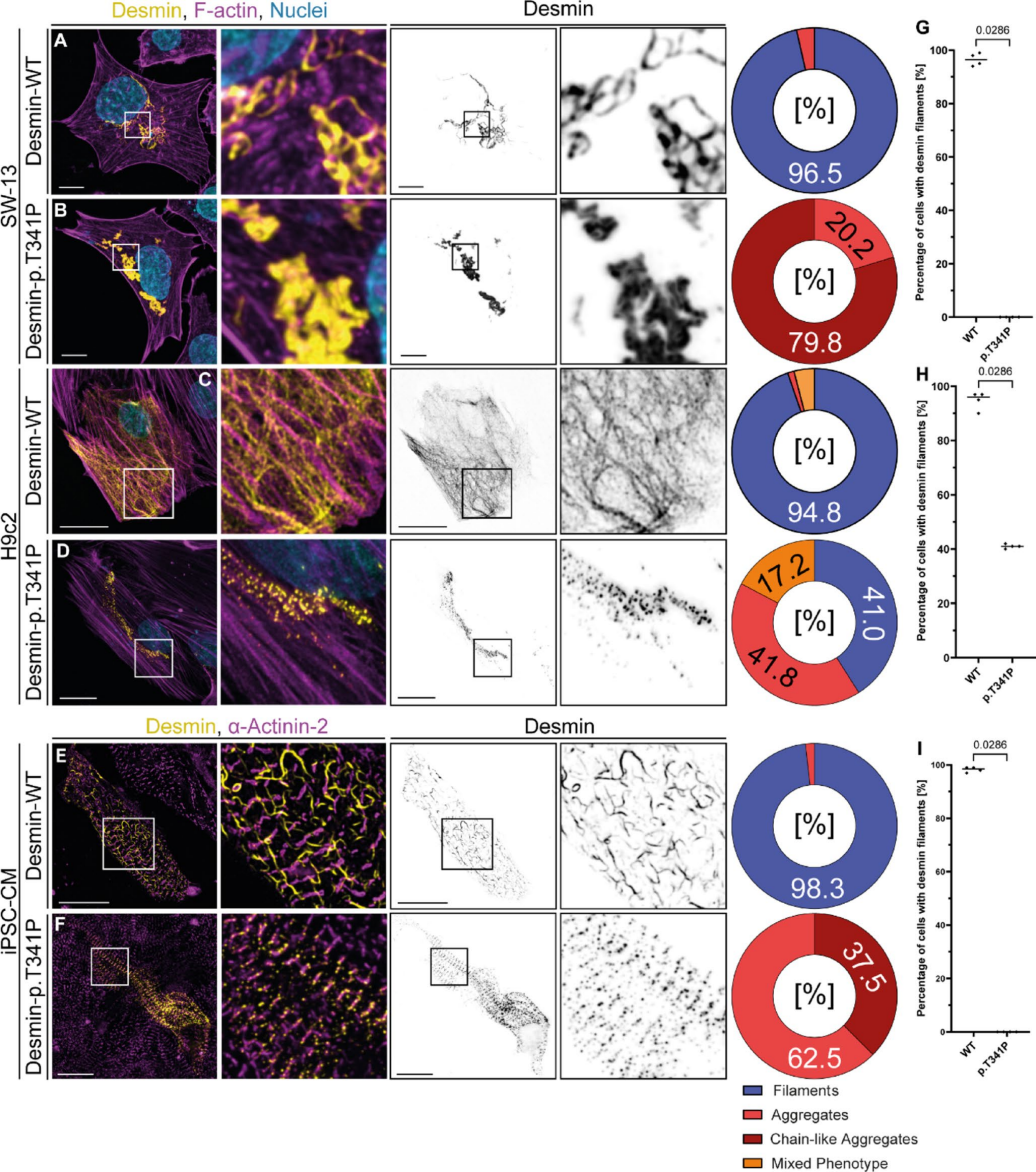
Cellular analysis of *DES*-p.T341P. Representative maximum intensity projections of SW-13 **(A-B)**, H9c2 cells **(C-D)** and cardiomyocytes derived from induced pluripotent stem cells **(E-F)** are shown. Scale bar = 10 μm (SW-13) or 20 μm (H9c2 and iPSC-derived cardiomyocytes). The different cellular phenotypes were quantified and are shown as pie and dot plots. For details of the four prototypes (‘Filaments’, ‘Aggregates’, ‘Chain-like aggregates’, and ‘Mixed phenotype’), see Figure S1 in the Supplementary Material. In total, four independent cell transfection experiments (*n* = 4) were performed and about 100 transfected cells were analyzed per transfection experiment. **(G-I)** The non-parametric Mann-Whitney test was used for statistical analysis (*p < 0.05) of independent transfection experiments (*n* = 4)

We started our functional analysis using transiently transfected SW-13 cells, since this cell line does not express endogenous desmin or any other cytoplasmic intermediate filament proteins [21]. Wild-type desmin assembled in this cell line into filamentous structures (Fig. 2A), whereas desmin-p.T341P formed aberrant cytoplasmic desmin aggregates, which are partially clustered or formed chain-like structures (Fig. 2B). We verified these findings in H9c2 cells (Fig. 2C-D). Similarly, desmin-p.T341P formed aberrant aggregates in iPSC-derived cardiomyocytes (Fig. 2E-F). A significant number of these aberrant aggregates associated with the Z-bands in iPSC-derived cardiomyocytes (Fig. 2F). We analyzed the fluorescence intensity of desmin-EYFP with a plate reader and in addition by FACS analysis (Figure S2A, Supplementary Material). These experiments revealed comparable expression levels of desmin-WT and -p.T341P (Figure S2B-D, Supplementary Material).

**Fig. 3:**
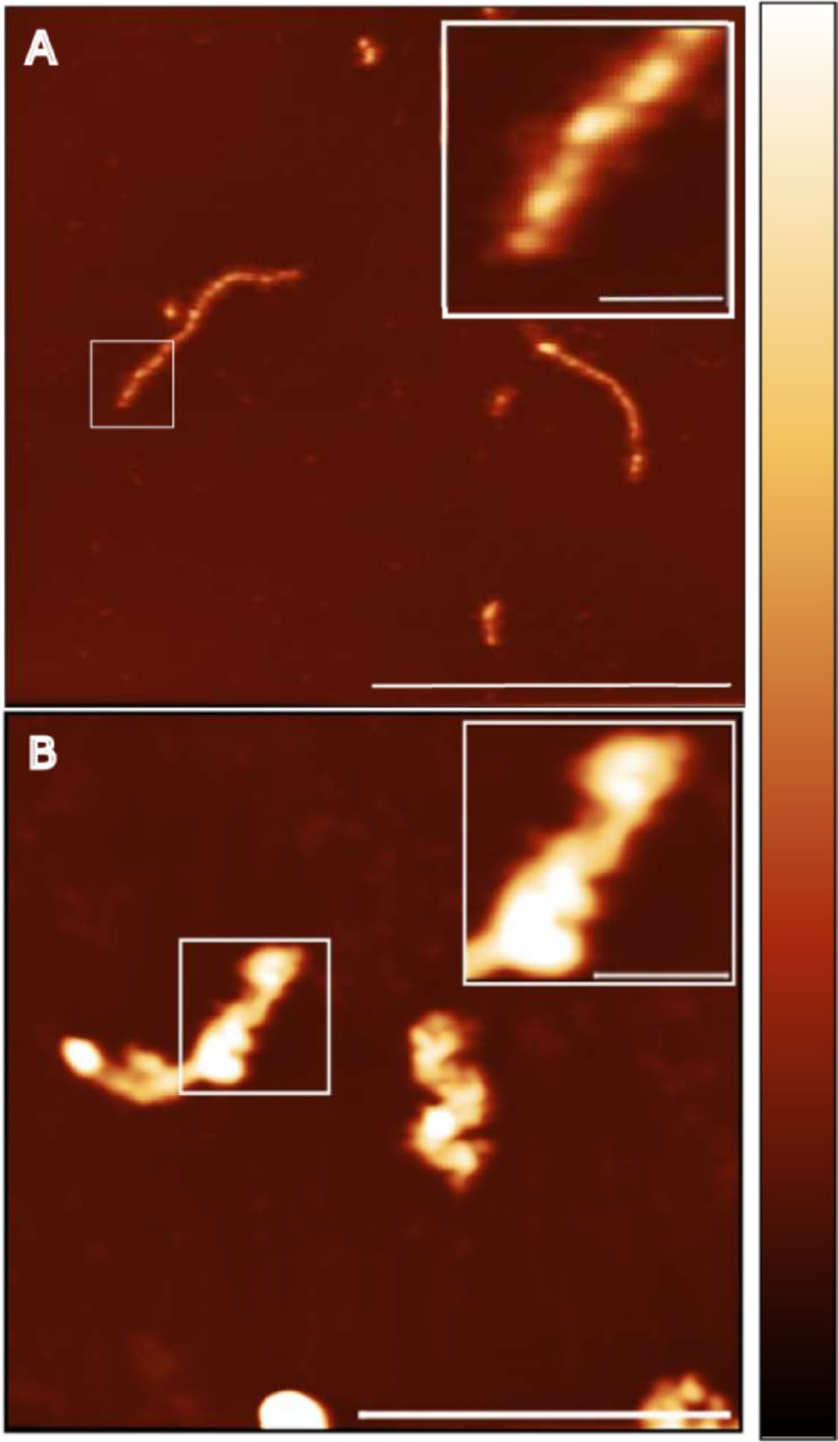
Representative atomic force microscopy topographic images of wild-type and mutant desmin (p.T341P). **(A)** Wild-type desmin assembled into long straight filaments with a typical length of approximately 600 nm. **(B)** Desmin-p.T341P formed aberrant filament-like structures. These structures were shorter and seem thicker than the regular wild-type desmin filaments indicating a borderline phenotype. Scale bars correspond to 1 μm **(A)** and 500 nm **(B)** in the overview and 100 nm in the insets, while the color bar corresponds to a height of 11 nm (wild-type) or 21 nm (p.T341P)

In addition, we expressed and purified wild-type and mutant recombinant desmin and performed likewise filament assembly assays using AFM analysis. Wild-type desmin assembled into typical long straight filaments (Fig. 3A), whereas desmin-p.T341P formed shorter and thicker aberrant structures (Fig. 3B).

## Discussion

A typical hallmark of pathogenic *DES* variants is an aberrant cytoplasmic desmin aggregation [17, 22]. However, it is currently difficult to predict the impact of novel *DES* missense variants on the filament assembly. Two highly conserved hotspot regions localized at the N-terminus of coil-1 [23, 24] and at the C-terminus of coil-2 [25] were recently recognized. Several missense variants in these regions cause desmin filament assembly defects. Recently, Pauls described the novel *DES*-p.T341P variant localized in the coil-2 subdomain in a small Russian family with MFM in combination with heart failure [12–14]. This variant cosegregated with the disease in this family [12], which is a supporting criterion according to the guidelines of the of the American College of Medical Genetics and Genomics (ACMG, PP1). Additionally, *DES*-p.T341P is completely absent in human genetic population databases (https://gnomad.broadinstitute.org/gene/ENSG00000175084?dataset=gnomad_r4 and https://rgc-research.regeneron.com/me/gene/DES, 2nd February 2026). According to the ACMG guidelines this is a moderate criterion (PM2) for pathogenicity [26]. Several in silico prediction tools support a deleterious effect of *DES*-p.T341P (PP3, Supplementary Table S1). In addition, the *DES* gene has a low rate of benign missense variants and most pathogenic variants in this gene are pathogenic missense variants [8] (PP2, ACMG). However, functional data to this specific variant are currently missing.

Therefore, we investigated here if the filament assembly of desmin-p.T341P is disturbed. We used different cell lines and cardiomyocytes derived from iPSC and investigated the filament assembly revealing an aberrant cytoplasmic desmin aggregation. However, most of the transfected cells formed in case of desmin-p.T341P cytoplasmic aggregates, which were partially connected and formed chain-like clusters or associated with the Z-bands. In a second approach, we used AFM to investigate the filament or aggregate formation of recombinant wild-type and mutant (p.T341P) desmin. These experiments revealed likewise aberrant desmin structures for mutant desmin-p.T341P, whereas wild-type desmin formed regular filaments. Recently, Brnich et al. provide recommendations about the functional analysis of genetic variants [27]. According to these recommendations, we showed that the desmin filament assembly could be used to classify *DES* variants [17]. Therefore, the damaging effect of desmin-p.T341P on the filament assembly is an additional moderate criterion for pathogenicity (PS3_moderate).

## Conclusion

In summary, our functional data showed a detrimental influence on filament formation of desmin-p.T341P (PS3_moderate, ACMG). In summary, *DES*-p.T341P fulfills two moderate (PM2 and PS3_moderate) and three supporting criteria (PP1, PP2 and PP3) for pathogenicity, which support the classification as a likely pathogenic variant according to the ACMG guidelines [26]. Therefore, functional analysis of the desmin filament assembly can contribute to classify the pathogenicity risk of novel genetic *DES* variants and may be helpful in genetic counselling of further patients with MFM or cardiomyopathies.

## Supplementary Information

Below is the link to the electronic supplementary material.

Supplementary Material 1

## Data Availability

All data are contained in this manuscript. The generated plasmids are available on request from the corresponding author.
