# Supplementary material for: The myofibrillar myopathy–linked variant DES-p.T341P impairs desmin filament assembly
Source: Mol Biol Rep. 2026 Mar 25;53(1):539. doi: 10.1007/s11033-026-11696-z (PMC13018087; doi:10.1007/s11033-026-11696-z)
Supplement: Supplementary file 1 — Supplementary Material 1 [file 11033_2026_11696_MOESM1_ESM.docx]

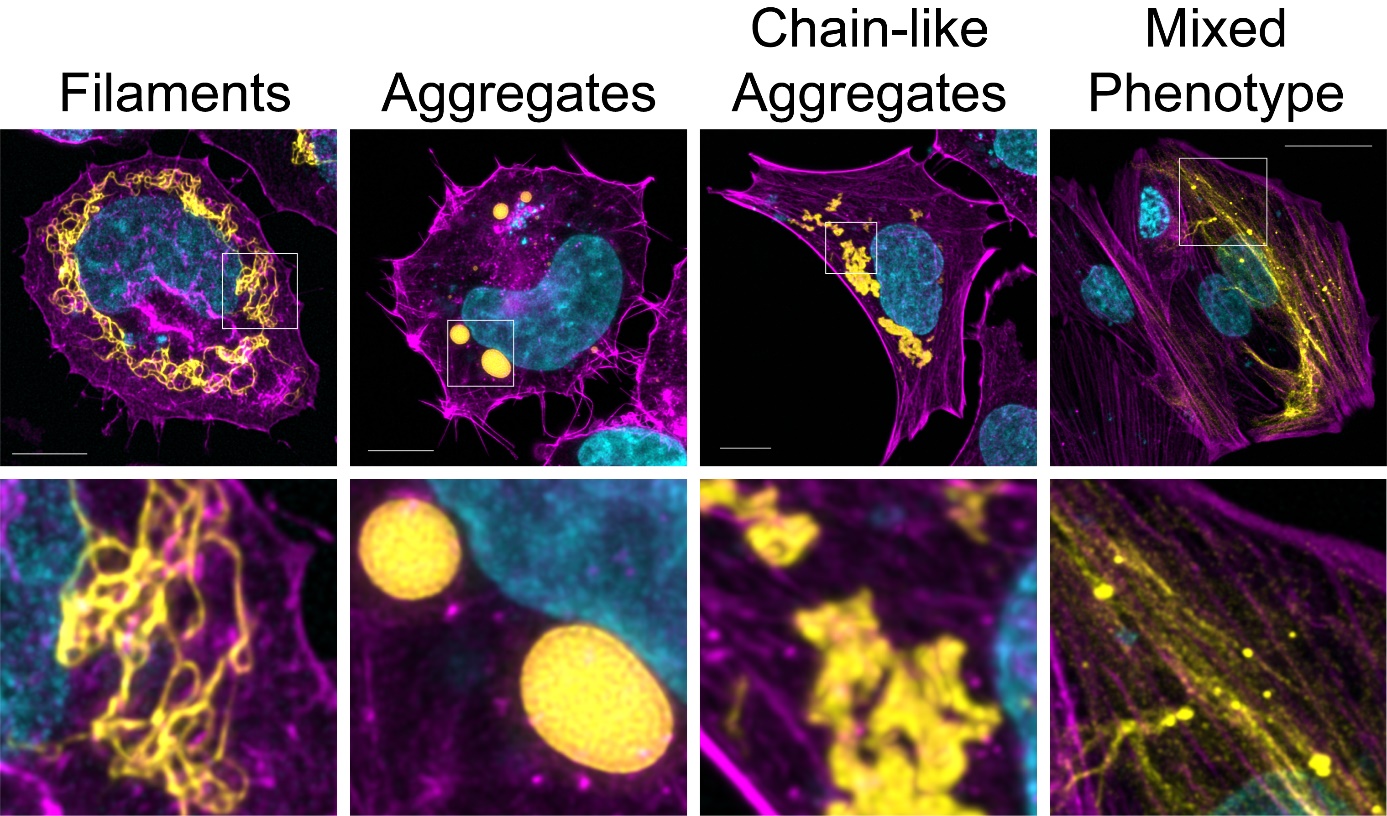


**Figure S1.** Four phenotypic cellular prototypes were defined. Cells containing exclusively desmin filaments of varying size and length (‘Filaments’); cells with cytoplasmic, isolated aggregates (‘Aggregates’); cells with ‘Chain-like Aggregates’ and cells displaying filaments and aggregates (“Mixed Phenotype”).


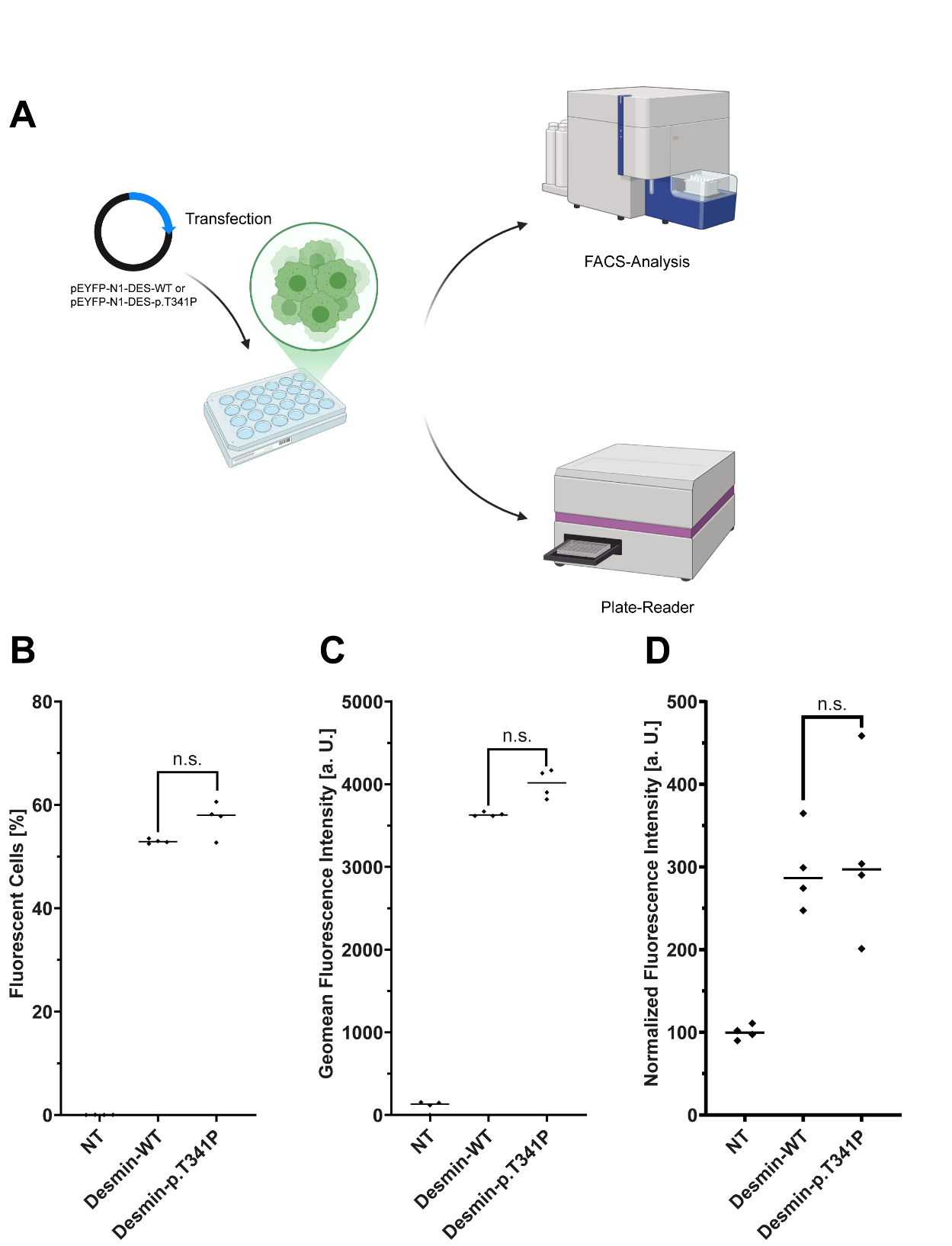


**Figure S2.** Expression analysis. **(A)** Desmin-EYFP expression analysis of transiently transfected cells was quantified by fluorescence activated cell sorting (FACS) and by fluorescence analysis using a plate reader. **(B)** Percentage of transiently transfected SW-13 cells expressing desmin-WT or -p.T341P. **(C)** FACS analysis. The geomean of the fluorescence intensity is shown in arbitrary units (a. U.). **(D)** Normalized fluorescence intensities of SW-13 cells (48 well plate, 1 cm² growth area) transfected with pEYFP-N1-DES-WT and pEYFP-N1-DES-p.T341P (n=4; n.s.= not signicifant, non-parametric Kruskal-Wallis test). NT= not transfected.

**Table S1.** *In silico* prediction of *DES*-p.T341P.

| Name of the variant | | ***DES*-p.T341P** (NP_001918.3) | |
| --- | --- | --- | --- |
|  |  | *DES*-c.1021A>C (NM_001927.4) | |
| Chromosomal position | | 2:219420951A>C | |
| Affected protein domain | | Rod domain (Coil-2) | |
| Database Information | ClinGen (Gene Disease Validity), 2^nd^ February 2026 | Dilated Cardiomyopathy – Definitive  Arrhythmogenic Right Ventricular Cardiomyopathy – Moderate  Myofibrillar Myopathy – Moderate Actionability | https://search.clinicalgenome.org/kb/genes/HGNC:2770 |
|  | OMIM (Online Mendelian Inheritance in Man), 2^nd^ February 2026 | Cardiomyopathy, dilated, 1I  Myopathy, myofibrillar, 1 | #604765  #601419 |
|  | gnomAD v4.1.0 (Minor Allele Frequency), 2^nd^ February 2026 | - | https://gnomad.broadinstitute.org/ |
|  | RGC Million Exome Variant Brower (Alternate Allele Frequency), 2^nd^ February 2026 | - | https://rgc-research.regeneron.com/ |
|  | ClinVar, 2^nd^ February 2026 | Not listed | https://www.ncbi.nlm.nih.gov/clinvar/ |
| *In Silico* Predictors (Individual Predictions) | AlphaMissense | Deleterious Moderate (0.965) | |
|  | REVEL | Deleterious Moderate (0.9) | |
|  | EVE | Deleterious (0.720) | |
|  | MutationTaster | Deleterious (1) | |
|  | DANN | Deleterious (1) | |
|  | MetaLR | Deleterious (0.92) | |
|  | BayesDel | Deleterious Moderate (0.33) | |
|  | SIFT | Uncertain (0.018) | |
|  | FATHMM | Uncertain (-3.82) | |
|  | Aggregated Prediction (<https://franklin.genoox.com>, 10^th^ February 2026) | Deleterious (0.87) | |
